# Learning biological network using mutual information and conditional independence

**DOI:** 10.1186/1471-2105-11-S3-S9

**Published:** 2010-04-29

**Authors:** Dong-Chul Kim, Xiaoyu Wang, Chin-Rang Yang, Jean Gao

**Affiliations:** 1 Department of Computer Science and Engineering The University of Texas at Arlington Arlington, TX, 76019, USA; 2 Simmons Comprehensive Cancer Center, The University of Texas Southwestern Medical Center, Dallas, TX, 75390, USA

## Abstract

**Background:**

Biological networks offer us a new way to investigate the interactions among different components and address the biological system as a whole. In this paper, a reverse-phase protein microarray (RPPM) is used for the quantitative measurement of proteomic responses.

**Results:**

To discover the signaling pathway responsive to RPPM, a new structure learning algorithm of Bayesian networks is developed based on mutual Information, conditional independence, and graph immorality. Trusted biology networks are thus predicted by the new approach. As an application example, we investigate signaling networks of ataxia telangiectasis mutation (ATM). The study was carried out at different time points under different dosages for cell lines with and without gene transfection. To validate the performance ofthe proposed algorithm, comparison experiments were also implemented using three well-known networks. From the experiment results, our approach produces more reliable networks with a relatively small number of wrong connection especially in mid-size networks. By using the proposed method, we predicted different networks for ATM under different doses of radiation treatment, and those networks were compared with results from eight different protein protein interaction (PPI) databases.

**Conclusions:**

By using a new protein microarray technology in combination with a new computational framework, we demonstrate an application of the methodology to the study of biological networks of ATM cell lines under low dose ionization radiation.

## Background

Bayesian networks are widely applied to a variety of domains such as business, engineering, and medicine [1]. The networks can be used to make optimal inference decisions based on Bayesian theory as well as to find the causal relationship between different entities as a graph model.

To perform an efficient inference and correct representation of the dependency relationship, an optimal structure is constructed to maximize the probabilistic fitness to the given data. Determining the optimal network through learning structures of Bayesian networks has been explored over the last decade, which contains the development of searching and scoring schemes. The searching is to find the structure that has the highest score among all possible ones. Since the searching space grows exponentially when the number of variables (nodes) increases, it is known as NP hard [2]. The scoring computes the score of a structure to evaluate how well it fits a given data.

Until now, several scoring functions have been developed including the well known Cooper-Herskovits scoring function as in K2 algorithm [3], the likelihood equivalence Bayesian Dirichlet (BDe) scoring function [4], and the minimum description length (MDL) scoring function [5]. In addition to serving as a scoring function, the K2 algorithm [3] functioning as a searching method has attracted attention from researchers due to its performance and efficiency till quite recently. However, the assumption of K2 algorithm is that the order of variables is correctly known. In other words, the performance highly depends on if the variables are well ordered. With regard to structure learning, Hill-Climbing greedy algorithm was used as a searching algorithm in [6]. Conditional independence property and mutual information were also employed for structure learning [7].

The goal of this study is to infer the proteomic signaling pathways affected by DNA damage, DNA repair, cell cycle checkpoints, and cell apoptosis under the influence of different radiation dosages. An emerging protein microarray technology, called the revers-phase protein microarray (RPPM), in conjunction with the quantum dots (Qdot) nano-technology, is used as the detection system. We study the proteomic responses at different time points (1h, 6h, 24h, 48h, and 72h) under different dosages (4 cGy, 10 cGy, 50 cGy, 1 Gy, and 5 Gy).

To infer the signaling pathways under different radiation dosages, in this paper we propose a new Bayesian network structure learning algorithm using the mutual information, conditional independence, and property of immorality in graph. Our method has two important features. First, the algorithm does not provide the direction for every edge in a predicted network. Since a signaling pathway is composed of successive and oriented interactions of molecules, even a small number of edges that have incorrect directions can cause significant effect in biological network analysis. To avoid a misleading result, therefore, we aim to report the most trusted edges, though a complete directed graph is not produced. Second, we focus on reducing wrong edges even though price for missing edges is paid. In other words, reliable, though not complete, information is reported as opposed to complete but uncertain information. To achieve these two goals, we initially exclude edges with low mutual information, and strictly carry out conditional independence test and immorality test for each candidate edge in order to remove incorrect edges. In the following sections, we first introduce the main steps of the proposed methodology. Then we use well known standard networks to evaluate the performance of the algorithm. Finally proteomic networks for ATM cell lines under different radiation dosages are presented.

## Methods

### Bayesian networks and MDL scoring function

Consider a finite set* V_n_* = {*X*_1_, *X*_2_, …, *X_n_*} of* n* discrete random variables for a given data set. With these variables, a Bayesian network consists of qualitative and quantitative parts. The qualitative component means that each variable can be a node, and* n* nodes can be connected by edges,* E_G_*. A Bayesian network is a* directed acyclic graph* (DAG),* G* = (*V_n_*, *E_G_*) which represents the conditional dependence between variables through oriented edges. The quantitative component of a Bayesian network contains a set of conditional probabilities, *p*(*X_i_*|*Pa*(*X_i_*)) for each variable* X_i_*. *Pa*(*X_i_*) is a set of variables which are the parents of* X_i_* in graph *G*. The joint distribution with these conditional probability distributions is defined as follows:(1)

Therefore, once we know the structure of a Bayesian network and the conditional probabilities of each node, we will know the joint probability distribution. The objective of this study is to infer the biological structure* G* that best matches the protein array measurement data *D*. Toward our goal, we propose a new searching strategy in this paper to find the best structure. The optimality of the current defined structure is evaluated using a scoring function based on MDL (minimum description length) [5,8]:

and
				(2)

where* r_i_* is the number of states for variable* X_i_*,* q_i_* is the number of possible configurations of the parent set Pa(Xi) of* X_i_* with , and* N_ijk_* is the number of instances in the data set* D* where the variable* X_i_* takes the value* X_ik_* and the set of variables* Pa*(*X_i_*) have the* j *th (*j *=1,2,…,*q_i_*) configuration in the parent set of* Pa*(*X_i_*). *N_ij_* is the total number of the *j *th configuration of* Pa*(*X_i_*) [9].

### Mutual information and conditional independence

In our proposed algorithm, mutual information (MI) is used to decide which edge is more significant than others. More precisely, we sequentially decide the connection and orientation of edges which is ordered by MI. Mutual Information between random variables X and Y is defined as follows:(3)

where* H*(*X*) is the entropy of* X*, and* H*(*X*|*Y*) is conditional entropy of* X* given* Y*. Entropy and conditional entropy are defined as;(4)

where* N* is the number of samples and* x_i_* is a state of variable* X*.

In our algorithm, conditional independence (CI) is also used to find which edge is incorrect in a triangular structure. CI is defined as follows:

*X_i_* and *X_j_* is conditionally independent given *X_k_* if *P*(*X_i_*,*X_j_*| *X_k_*) = *P*(*X_i_*|*X_k_*)*P*(*X_j_*|*X_k_*) (5)

Therefore, once the edge we consider to connect makes a new triangle, we can test (5) for all three edges of the triangle. Based on the result of the CI test, we can update the network.

### Property of equivalence class and immorality

**Figure 1:**
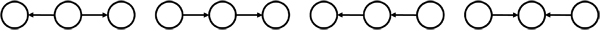
Structures with three nodes and two edges

In searching and scoring scheme for learning structure of Bayesian networks,* equivalent class* should be considered [10,11]. This means when more than two graphs are equivalent, those graphs have the same dependency; therefore, two structures have identical scores. As an example, two variables* A* and* B* may have two different structures as* A → B* or* A ← B*, however, as equivalent classes, these two structures end up having the same score for any given data. For a three nodes, two edges structure shown in Figure 1, suppose the first three structures have the same score and are equivalence class. For the last one with a different score, we call this kind of structure (head to head)* immorality*. So, based on the different scores from the equivalence class and immorality, we can decide the direction of two edges in searching algorithm. This is because immorality structure normally has higher score than other structures in the case of three nodes and two edges if original relationship of three nodes is immorality. However, if the score with immorality structure is lower than any other structures, we cannot decide the direction of the edges.

### Algorithm

The proposed algorithm initially starts from a non-connected network in which there is no edge between nodes. We calculate MI for two nodes of all edges, and the edges whose MI is less than threshold* α* are excluded from candidates of correct edges. In this paper,* α* is heuristically chosen as 0.0001. Based on these MI values, edges are ordered. After finishing the ordering, we sequentially decide the connection for each edge in the MI order. Since earlier decided edges can affect the decision for other edges, we choose edges which have higher chances to be correct edge as it is supposed that higher MI has higher probability of connection. Unlike greedy searching algorithms that normally involve many times of iterations, we test each edge one time only. Before we decide the connection edge by edge, each edge is categorized into three cases which have different decision rules as discussed in the following paragraphs. 

The first case is when the* current edge* creates a triangular structure. Current edge indicates an edge currently to be decided in the algorithm. In this case, as we noted earlier, CI test is performed to find which edge is not necessary among the three edges of the triangle. For instance, suppose there are three nodes *i*,* j*, and* k*, and the edge* E*(*i*,* j*) is the current edge. Since* E*(*i*,* j*) creates a triangle structure, we test CI of three edges,* E*(*i*, *j*),* E*(*j*,* k*), and* E*(*k*,* i*). If only one pair of nodes is conditional independent, the edge between the two nodes is deleted. The other results of CI test are ignored which means we do not use current edge. After one edge is deleted or current edge is abandoned, we perform Immorality test with two remained edges in order to find the direction of them.

The second case is that the current edge creates a cycle in the graph which means there should be at least more than one Immorality in the cycle because Bayesian Network is an acyclic graph. Since most of the edges with relatively low MI creates cycles and are added after correct network is constructed already, we have to avoid the wrong edges with immorality test between current and other linked edges. If there is no immorality, we do not use the current edge. As an example, given a structure with four nodes, *h*, *i*,  *j*, *k* and the current edge as* E*(*i*,* j*), if* E*(*i*, *j*) makes a new triangle structure, we perform two immorality tests with two pairs of edges like* E*(*i*,* j*),* E*(*h*, *i*) and *E*(*i*, *j*), *E*(*j*,* k*). If both of the two pairs have immorality, we choose one randomly. However, in most cases, only one of the two pairs has immorality. In our example, if *E*(*i*,* j*) and* E*(*h*,* i*) has immorality but not* E*(*i*,* j*) and* E*(*j*,* k*), we connect the current edge* E*(*i*,* j*) and give the head to head direction like* h→i←j*.

**Figure 2:**
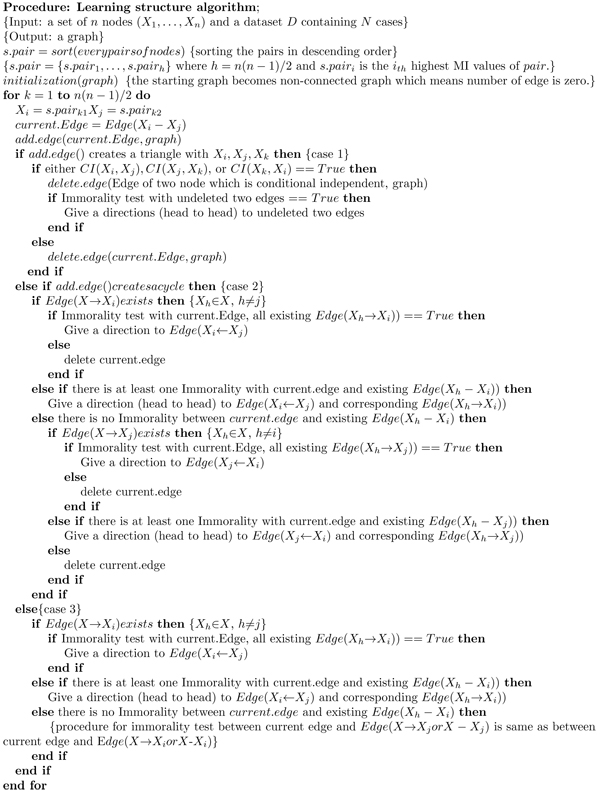
Pseudo code for suggested algorithm

The third case is all other cases except the aforementioned two cases. In this situation, we connect current edge with other edges without any test except MI test for the orientations of edges. The pseudo code for our proposed algorithm is outlined in Figure 2.

## Results and discussion 

### Algorithm evaluation

**Figure 3:**
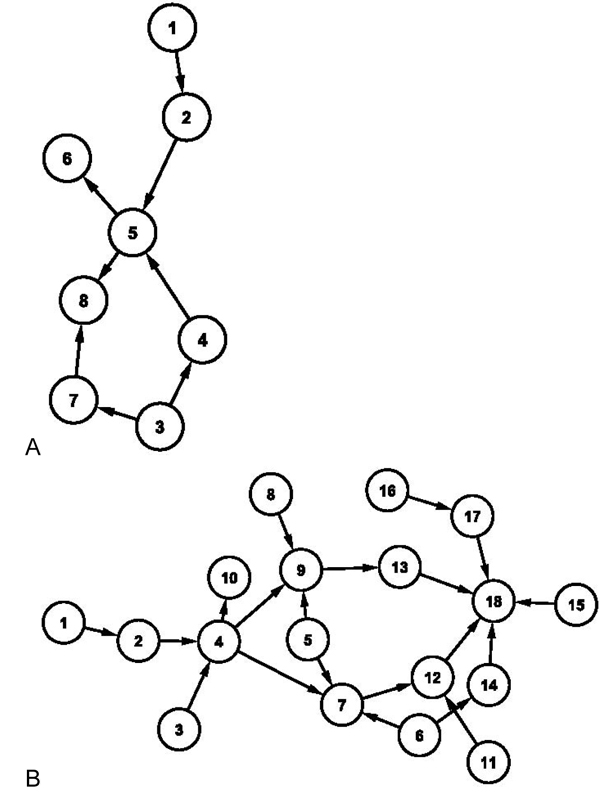
ASIA and CAR DIAGNOSIS2 networks. ASIA network has 8 nodes and 8 edges (A), and CAR DIAGNOSIS2 network has 18 nodes and 20 edges (B).

**Figure 4:**
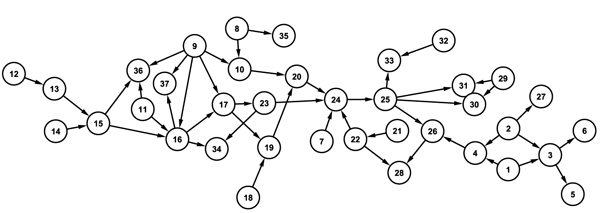
ALARM network. ALARM network has 37 nodes and 46 edges.

To evaluate the algorithm, we adopted three well-known networks, ASIA [12], CAR DIGNOSIS2(Brent Boerlage, http://www.norsys.com), and ALARM [13] (Figures 3 and 4). With these networks, we created 20 dataset for each network using TETRAD4 (http://www.phil.cmu.edu/projects/tetrad/), and each dataset has 50,000 cases (samples) based on the structure and conditional probability of three networks. To avoid the bias of predefined correct node order in which hierarchically higher node has front order of node number when data is generated, we randomly assign the order of node. The new algorithm is performed using the generated data, and then we compare the predicted network to true network. To see how well the suggested method can predict the network, parameters including missed edges (ME), wrong edges (WE), correct edges in trustworthy network (CETN), number of edges of trustworthy network (NETN) are calculated. ME is the number of true network's edges that the algorithm couldn't find. Since our result network does not have orientation in every edge, the number of wrong orientation edges is not available to be compared because we focus on providing the most confident edges. WE means the edges the algorithm found do not exist in the true network. CETN indicates that the edges in TN are the same as edges of the true network with correct orientations. NETN refers to the number of edges of a trustworthy network. For the performance comparison, we compare the results of the new algorithm with other structure learning algorithms including the hill-climbing searching method with MDL scoring function and the well known K2 algorithm with random ordered nodes.

### Results of structure learning for known networks

ASIA network has 8 nodes and 8 edges. Since ASIA network is a small size graph, the predicted network by our method does not have any WE and even ME is just 0.1 on average as shown in Table 1. CAR DIAGNOSIS2 network consists of 18 nodes and 20 edges as a moderate size network. The suggested algorithm satisfactorily achieves 0.8 WE, but ME is increased to 2 which is shy of the result by K2 algorithm but superior to hill-climbing (Table 2). ALARM network has 37 nodes and 46 edges. The average number of 3.83 for WE is far below the other two methods. Similarly to the CAR DIAGNOSIS2 network, the ME measurement of the proposed algorithm is worse than other methods (Table 3). In addition, Table 4 presents the accuracy of trustworthy network. Although the accuracy is decreasing when the network size increases, the result shows more than 90% accuracy in all of three networks.

**Table 1:** Result for the Asia network

Method	ME	WOE	WE
Our Method	0.1	n/a	0
Hill-Climbing	2.2	0.8	4.8
K2	1	3.45	4.8

**Table 2:** Result for the Car Diagnosis2 network

Method	ME	WOE	WE
Our Method	2	n/a	0.8
Hill-Climbing	2.35	5.9	8.4
K2	1.4	9.4	16.3

**Table 3:** Result for the Alarm network

Method	ME	WOE	WE
Our Method	6.05	n/a	3.85
Hill-Climbing	1.55	9.75	9.4
K2	2.05	22.5	53.75

**Table 4:** Result for Trustworthy Network

Network	NETN	CETN	ACCURACY
ASIA	4	4	100%
CAR DIAGNOSIS2	13.8	13	94%
ALARM	29.7	26.8	90%

### Learning structure of pathway in ATM cell

We applied quantum dot reverse-phase protein microarray to profile the dynamic responses of several signaling pathways, including DNA damage, DNA repair, and cell cycle checkpoints, under ionizing radiation (IR). Ataxia telangiecstasia mutation-deficient (ATM-) and -proficient (transfected with full length ATM construct, ATM+) cells were treated with different doses of IR and cell lysates were collected at different time-points, serially diluted and spotted on an array in triplicate. The intensities of all antibodies were normalized relative to those of control and were normalized to values from zero to one. The arrays were then probed with specific antibodies. 67 antibodies have been evaluated for the dynamic change of the network. The complete list of the antibodies is shown in Table 5. The five applied IR doses are 4 cGy, 10 cGy, 50 cGy, 1 Gy, and 5 Gy. Both types of cells for each dosage were observed at 1 h, 6 h, 24 h, 48 h, and 72 h.

**Table 5:** 67 antibodies used in the reverse-phase protein array for ATM radiation study

0	1	2	3	4	5	6	7
mTOR	b-catenin	Chk1	E-Cad	MDM2	p38	p-p38	pChk2
8	9	10	11	12	13	14	15
pATM	Rb	pRb	Raf-1	p-Src	PTEN	STAT3	Caspase8
16	17	18	19	20	21	22	23
IGF1-R	IRS-1	GSK3ab	pGSK3ab	pMDM2	pSTAT3	AKT	pAKT
24	25	26	27	28	29	30	31
Caspase3	DNAPK	pDNAPK	EGFR	pEGFR	NFkBp65	pNFkB	NQO1
32	33	34	35	36	37	38	39
p21	p27	p-PTEN	pRaf1	Bcl-2	pBcl-2	Caspase9	cdk4
40	41	42	43	44	45	46	47
pErk	lkBa	plkBa	JNK	Klotho	p16	p53	p-p53
48	49	50	51	52	53	54	55
Smad3	Src	Vimentin	sClu	ATM	Chk2	Erk	HSP27
56	57	58	59	60	61	62	63
IGFBP	pChk1	pDNAPK	gH2AX	pIGF1-R(y1158.62.63)	pIGF1-R(y1162.63)	pIRS(Y896)	pIRS(Y1179)
64	65	66					
pJNK	p-mTOR	pSmad3					

The expression data is normalized with respect to Actin concentration on each microarray chip. The expression level of each antibody is discretized into 2 to 4 values using minimum entropy based discretization. For each IR dose, we have a total number of 30 samples for ATM+ and ATM- from the triplicate at different times. Among the 67 antibodies involved in the RPPM data, we select the most distinguishing ones between ATM+ and ATM- using a feature selection method developed in our early work [14].

**Figure 5:**
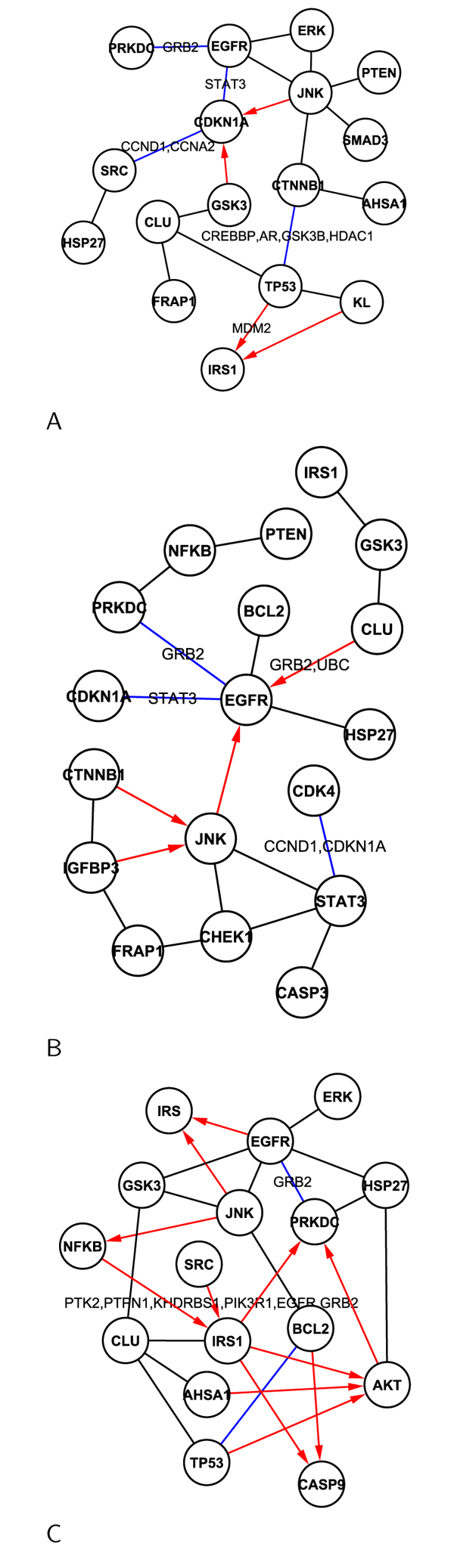
Signal networks under the dosages of 4cGy (A), 10cGy (B), and 50cGy (C).

**Figure 6:**
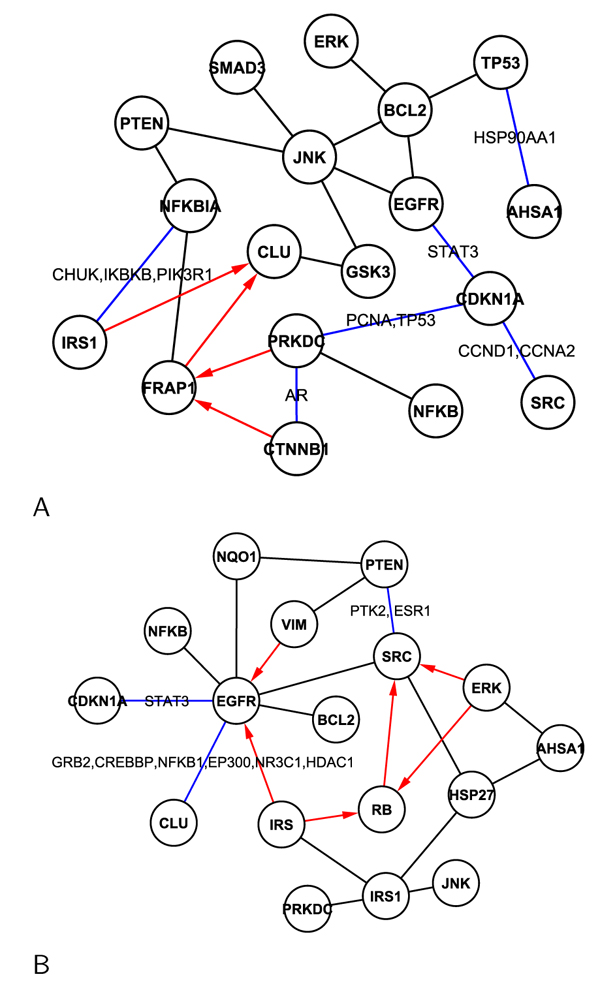
Signal networks under the dosages of 1Gy (A) and 5Gy (B).

Figures 5 and 6 show the discovered networks for the selected distinguishing antibodies under each IR dosage treatments. To validate the discovered networks, we compared our discovery with protein-protein interactions (PPI) results in eight different databases which are curated by experts based on biological experiments or reported literature (BIND, BIOGRID, Cognia, DIP, INTACT, Interactome studies, MINT, and MIPS). Since an edge in a discovered network means a cause and effect relationship and may not be direct interactions , additional components may exist between the two nodes that the edge connects. We referred to indirect interactions (one hop path) such as* Protein-Protein-Protein* from the PPI databases. In the discovered networks, red edges are trustworthy network. The red lines are superior to other colored lines. The black edges are found by our method only, while the blue solid lines indicate paths from PPI databases. The proteins over a blue path are the possible protein in one hop path between the two nodes. If there is no protein on blue line, it is a direct PPI. We can see the overlap and difference between the two approaches.

## Conclusions

Understanding the proteomic network structure reveals the inherent biological information flow which will lead to more effective therapies and disease treatments. In this paper, by using a new protein microarray technology in combination with a new computational framework, we demonstrate an application of the methodology to the study of biological networks of ATM cells under ionization radiation. Different networks were found through this study. The same technology can be extended to different biological problems. For future work, we intend to validate our discovery by carrying out biological experiments.

## Authors' contributions

Dongchul Kim and Jean Gao contribute the computational algorithm design and the manuscript writing. Xiaoyu Wang carried out the biological experiment for the RPPM data generation. Chin-Rang Yang is responsible for the overall project layout and direction.

## Competing interests

The authors declare that they have no competing interests.
